# Four-dimensional CT as a valid approach to detect and quantify kinematic changes after selective ankle ligament sectioning

**DOI:** 10.1038/s41598-018-38101-5

**Published:** 2019-02-04

**Authors:** Luca Buzzatti, Benyameen Keelson, Jildert Apperloo, Thierry Scheerlinck, Jean-Pierre Baeyens, Gert Van Gompel, Jef Vandemeulebroucke, Michel de Maeseneer, Johan de Mey, Nico Buls, Erik Cattrysse

**Affiliations:** 1 Department of Physiotherapy, Human Physiology and Anatomy (KIMA), Vrije Universiteit Brussel, Brussel, Belgium; 2 Faculty of Medicine and Pharmacy, Vrije Universiteit Brussel, Brussel, Belgium; 3 Department of Radiology, Universitair Ziekenhuis Brussel, Brussel, Belgium; 4 Department of Orthopaedic Surgery and Traumatology, Universitair Ziekenhuis Brussel, Vrije Universiteit Brussel, Brussel, Belgium; 5 Department of Electronics and Informatics (ETRO), Vrije Universiteit Brussel, Brussel, Belgium; 6 Imec, Leuven, Belgium; 7 Department of Movement and Sport Sciences, Vrije Universiteit Brussel, Brussel, Belgium; 8 University College of Physiotherapy THIM, Landquart, Switzerland

## Abstract

The objective of the current study was to explore the potential of dynamic computed tomography to detect kinematic changes, induced by sequential sectioning of the lateral collateral ligaments of the ankle, during full motion sequence of the talocrural joint. A custom-made device was used to induce cyclic controlled ankle inversion movement in one fresh frozen cadaver leg. A 256-slice CT scanner was used to investigate four different scenarios. Scenario 1 with all ligaments intact was first investigated followed by sequential section of the anterior talo-fibular ligament (Scenario 2), the calcaneo-fibular ligament (Scenario 3) and posterior talo-fibular ligament (Scenario 4). Off-line image processing based on semi-automatic segmentation and bone rigid registration was performed. Motion parameters such as translation, rotational angles and orientation and position of the axis of rotation were calculated. Differences between scenarios were calculated. Progressive increase of cranio-caudal displacement up to 3.9 mm and flexion up to 10° compared to Scenario 1 were reported. Progressive changes in orientation (up to 20.6°) and position (up to 4.1 mm) of the axis of rotation were also shown. Estimated effective dose of 0.005 mSv (1.9 mGy CTDI_vol_) was reported. This study demonstrated that kinematic changes due to the absence of ligament integrity can be detected with 4DCT with minimal radiation exposure. Identifying abnormal kinematic patterns could have future application in helping clinicians to choose patients’ optimal treatment. Therefore, further studies with bigger *in vitro* sample sizes and consequent investigations *in vivo* are recommended to confirm the current findings.

## Introduction

Four-dimensional computed tomography (4DCT) is an increasingly popular imaging modality and in the last decade, its applications for musculoskeletal (MSK) investigations have become more apparent^1–3^. Within this modality, one of the main applications reported so far is the estimation of integrity of (intra-)articular ligaments and the analysis of complex motion in several joints with strong rotatory components^4^.

Considering the talocrural joint, a commonly used method to evaluate lateral collateral ligament integrity is anterior drawer and talar tilt stress radiography^5^. However, this technique has some limitations. A wide range of reported norm values makes the interpretation of this technique difficult. Also, the joint stress is normally produced by an examiner which makes this setup less reproducible and exposes the examiner to unnecessary radiation^6^. It was also seen that positional changes on stress radiographs were significantly lower compared with a 3D measurement system^7^. This may underestimate the true magnitude of these changes which could influence clinical decision making.

4DCT may be an alternative for such evaluations, as it provides 3D measurements over time. The dynamic evaluation of joint kinematics makes it possible to evaluate motion trajectory and patterns, and therefore could lead to identifying pathologies which may occur only during motion. The potential feasibility of 4DCT for such purposes is already shown in several studies^8–10^. Studies attempting to simulate pathological conditions showed that 4DCT may be able to detect altered joint kinematics. Simulation of subtalar joint instability by means of subsequent sectioning of the cervical ligament and the interosseous talocalcaneal ligament showed increases in joint motion amplitude^8^. In a similar study where the scapholunate ligament was cut to mimic scapholunate instability, orthopaedic surgeons could discern images before and after induced ligamentous damage. After ligament section, the movement of the scaphoid increased up to 1.39 mm compared to its original position. These findings support the added value of 4DCT for diagnosing carpal instabilities associated with ligamentous damage where static imaging techniques fail^9,10^.

4DCT was also shown to be reliable for joint kinematics analysis, as errors of less than 1° and 1 mm regarding rotation and translation values have been reported^11,12^. Moreover, two wrist studies showed strong inter-observer reliability for anteroposterior interval difference measurements in the pisotriquetral joint and scapholunate diastasis (ICC = 0.6–0.85)^13,14^.

Consequently, there is a body of evidence suggesting that 4DCT may be of value in musculoskeletal diagnosis and evaluation of musculoskeletal interventions. However, the capability and reproducibility of 4DCT to detect differences in motion is still under investigation. So far, no studies concerning 4DCT analysing the talocrural joint (patho)kinematics have been reported. Therefore, the objective of this study was to explore the potential capacity of 4DCT to detect such changes during full motion sequence of the talocrural and subtalar joint, provoked by sequential sectioning of the lateral collateral ligaments of the ankle.

## Materials and Methods

### Specimen

One fresh frozen right leg (Female – no age available due to privacy restriction policy) was used for this study. The specimen was allowed to slowly defrost for two days to preserve optimal tissues condition. To preserve the attachments of the tendons, the specimen’s lower limb was cut above the distal third of the thigh. Two holes were drilled through the proximal tibia to guarantee easy passage of the fixation pins. The tibial tuberosity was chosen as the reference marker for placing the vertical pin. One of the authors, with more than 20 years’ experience in teaching anatomy and human dissection, identified and marked the relevant ligaments.

Four different scenarios were investigated. Initially, movement with the lateral compartment of the ankle intact was studied (Scenario 1). Subsequently, the movement after consecutive sectioning of the anterior talo-fibular ligament (ATFL) (Scenario 2), the calcaneo-fibular ligament (CFL) (Scenario 3) and the posterior talo-fibular ligament (PTFL) (Scenario 4) were investigated. Approval from the medical ethical review board from the university hospital UZ Brussels was obtained before the start of this study (B.U.N 143201630099). All methods were performed in accordance with the relevant guidelines and regulations. The cadaver was legally donated and either the subject, or their next of kin, consented to the use of the body for research purposes.

### Experimental setup

**Figure 1:**
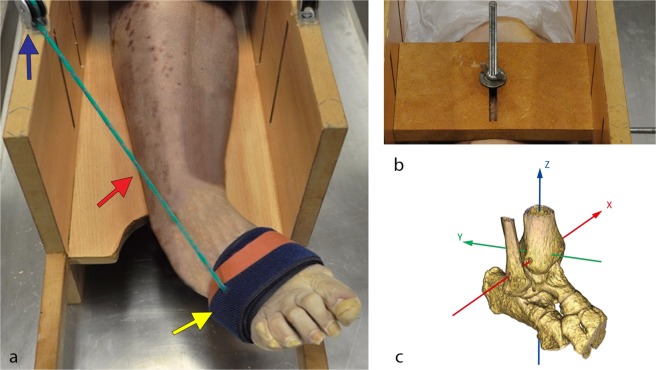
(**a**) Setup: pulley (blue arrow), cable (red arrow) and rigid band (yellow arrow); (**b**) Fixation pins; (**c**) reference frame. X axis: passing through the most prominent aspect of the medial malleolus, pointing from lateral to medial. Dorsiflexion-plantarflexion/medio-lateral translation; Y axis: perpendicular to Z and X axis. Pronation-supination/anterior-posterior translation; Z axis: passing through the Tibial Tuberosity. Abduction-adduction/cranio-caudal translation.

A custom-made wooden device (Fig. 1a) was used by one of the researchers to manually induce movement of the ankle at a pace of 25 cycles per minute. The device simulated the ankle movement from maximum dorsiflexion to full inversion (combined movement of plantar-flexion, adduction and supination) and back. Two pins (Fig. 1b) through the tibia fixed the specimen onto the device which was then locked onto the CT table to prevent unwanted movement during acquisition. The forefoot was secured with a rigid band attached to a cable which passed through a pulley ensuring consistent direction of the pulling force (Fig. 1a). No estimation of the force generated during the traction was performed. Four repetitions were performed to check repeatability and consistency of the motion prior to ligament sectioning.

While one researcher was manually generating the movement, a second researcher initialized and performed the scanning acquisition. Adequate protections were taken to ensure minimal exposure of the researcher to scatter radiations.

Four dynamic acquisitions were performed for each scenario to obtain a total of 16 dynamic data sets.

### CT protocol and Image processing

A wide beam CT (256-slice, GE Revolution Healthcare) was used for dynamic axial acquisition with the following parameters: 80 kVp, 25 mA, 0.14 s time resolution with half reconstruction, 120 mm z-axis coverage and 1.25 mm slice thickness. Each scan was triggered manually and had a continuous acquisition time of 3.92 s (cine mode). Iterative reconstruction was used and the estimated effective dose resulted in 0.005 mSv (1.9 mGy CTDIvol).

**Figure 2:**
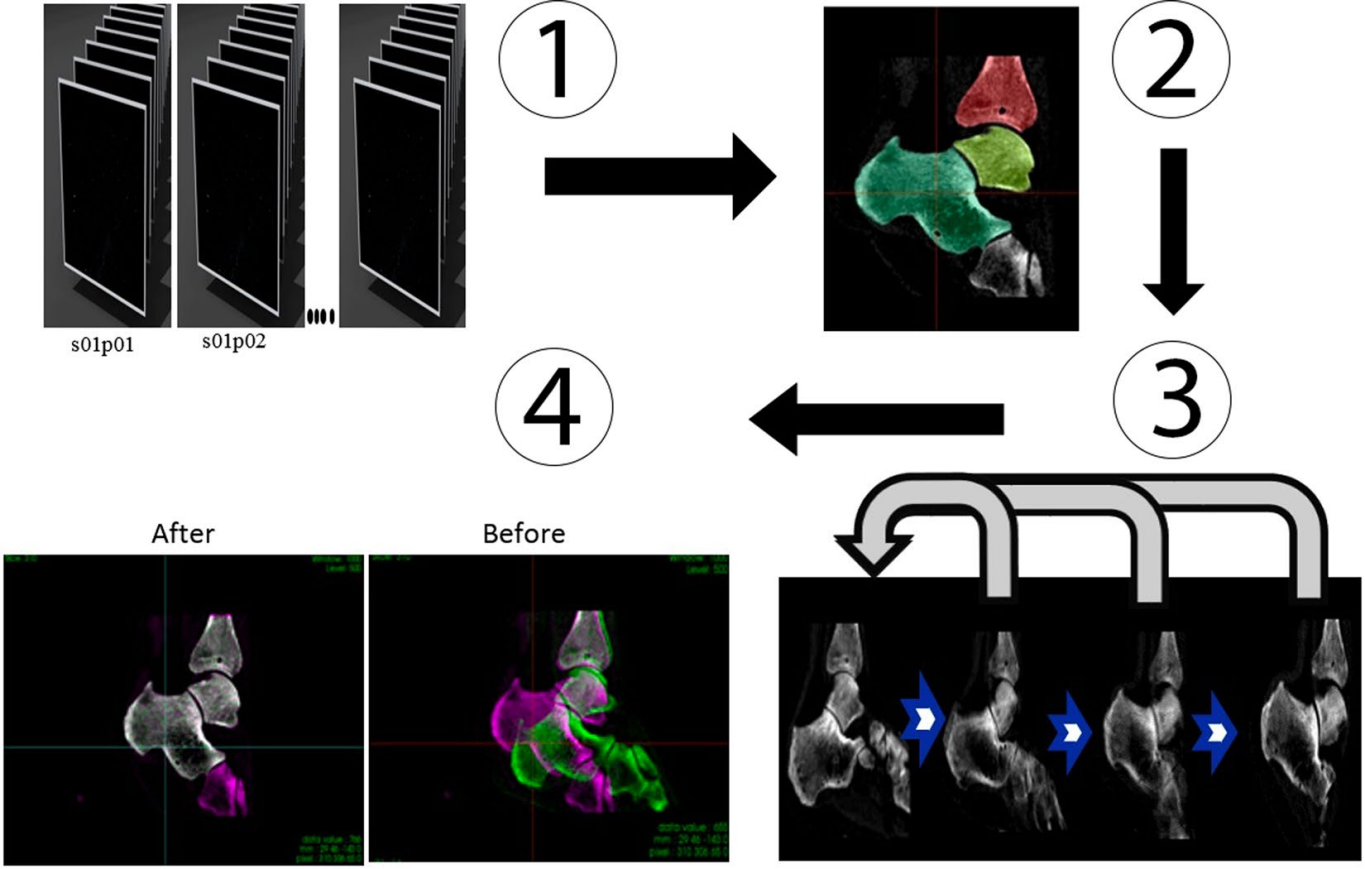
Workflow of the image processing. (1) Dynamic sequence obtained from CT acquisition; (2) Semi-automatic segmentation of the individual structures of interest in the reference image (static image) – Used as Region of interest for the registration; (3) Each individual structure of the reference image was rigidly registered to its corresponding structure in the moving image; (4) Images before and after registration. Pink represents the reference image and green the moving image. When pixels of the two images match, they display the colour grey. After the registration; Tibia, Talus and Calcaneus of the two images are perfectly aligned.

A total of 18 3D DICOM images, corresponding to phases of the motion from maximum dorsiflexion to full inversion, were selected and processed to form a 4D image dataset (dynamic 3D volume dataset of the motion). The processing was performed according to a fixed workflow (Fig. 2) using a C++ code based on the Insight Segmentation and Registration Toolkit (ITK)^15^. The talus and tibia were semi-automatically segmented using ITK SNAP^16^ followed by morphological operations and manual refinement. The segmentation was performed only on the “reference image” depicting the ankle at maximum dorsiflexion (start of the considered motion). The segmented bones served as masks for the registration process which can be described as an optimization problem over the parameters μ of the spatial transformation ${{\mathscr{T}}}_{\mu }$.

1 $$\hat{\mu }=arg\mathop{{\rm{\min }}}\limits_{\mu }\,{\mathscr{C}}(f(x),g(({{\mathscr{T}}}_{\mu }(x))))$$

The registration is guided by minimizing a cost function ${\mathscr{C}}$ (similarity metric). f and g represent the fixed and moving images respectively and *x* represents the spatial coordinates over the image domains.

Using mutual information as the similarity metric, the two bones in the remaining dynamic sequence (moving images) were rigidly registered to their corresponding bones in the reference image dataset (time-point 0). The rigid registration was performed using Elastix, an open source software (http://elastix.isi.uu.nl/), based on ITK^17,18^. The registration process resulted in 17 transformation matrices which aligned each of the moving images to the reference image. A set of corresponding points between the reference image and the moving image was obtained using 3D Scale Invariant Feature Transform (SIFT)^19^. These points were then used to estimate the accuracy of the registration process by computing target registration error (TRE)^20^.

### Kinematic parameters

The longitudinal axis of the tibia passing through the Tibial Tuberosity was aligned with the Z-axis of the CT. The X-axis, perpendicular to the Z-axis, passed through the most prominent aspect of the medial malleolus which was positioned at a height of zero along the Y-axis. This procedure, as well as the fact that the CT was zeroed in that position before acquisition, allowed use of the technical reference frame of the device for kinematic calculation of the talus within a functional anatomical approach. The orientation of the reference frame is shown in Fig. 1b. The positioning was guided by the scanner’s on-board lasers.

Transformation matrices obtained from the registration were used to calculate three different kinematic parameters to describe talocrural joint motion:

- displacement of the centre of the trochlea tali;
- rotation angles of the talus relative to the tibia;
- the study of the axis of rotation of the talocrural joint expressed as the finite helical axis (FHA).

The angles were extracted from the rotational components of the transformation matrix and they were based on ZYX Cardan conventions. Displacement was defined as the difference between coordinates of the centre of the trochlea tali at time-point 0 and the transformed coordinates at different time points. FHA was computed as suggested by Spoor and Veldpaus^21^.

### CT data analysis

Differences between the four repetitions were explored to investigate repeatability of the setup. Differences between Scenario 1 and the other scenarios were reported and plotted using mean values of the four repetitions for each time point. Changes of the axis of rotation were reported using orientation and position of the FHA. The axes were visualized through combined 2D graphical representations on the XY, XZ and YZ plane. Position of the FHA was defined as the intersection of the axis with a plane (parallel to the ZY plane) passing through the centre of the trochlea tali.

## Results

Four acquisitions were collected for each scenario. However, only one acquisition for Scenario 3 was available because of data transfer failure from the CT server to the archive.

Regarding quantitative evaluation of the registration accuracy, the average TRE over all scenarios was 0.42 mm (range: 0.33–0.5 mm).

### Repeatability of the setup

**Figure 3:**
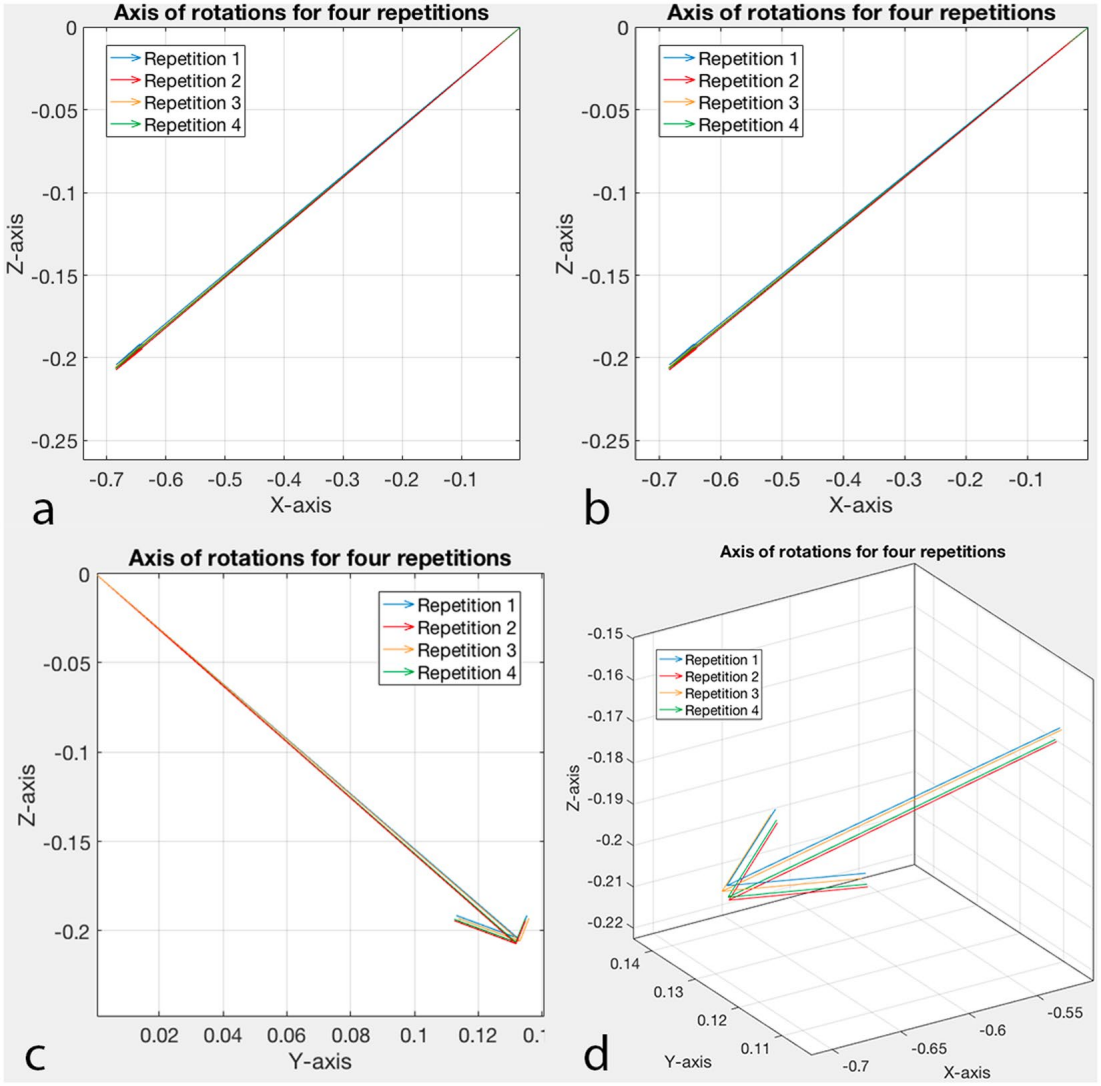
Axis of rotation orientation of four repetitions for Scenario 1 (Intact). (**a**) Orientation on the YZ plane; (**b**) orientation on the XZ plane. (**c**) Orientation on the XY plane. (**d**) Enlargement showing the axes of rotation of each repetition.

Between the four repetitions of Scenario 1, the maximum differences for the talocrural joint rotational components were 0.81°, 0.37° and 0.89° around the X-, Y- and Z-axis respectively. For the components of the displacement vector, these maximum differences were 0.27 mm, 0.66 mm and 0.08 mm respectively. The axis of rotation, defined by the FHA, presented a consistent orientation during the four repetitions (Table 1 and Fig. 3).

**Table 1:** Rotation angles, translations and axis of rotation for Scenario 1 (Intact).

Rotation angles (deg°)			
**Rep**	**X**	**Y**	**Z**
1	37.81	−1.97	12.36
2	37.81	−1.80	12.18
3	37.78	−1.88	12.19
4	37.00	−1.60	11.47
**Translations (mm)**			
**Rep**	**X**	**Y**	**Z**
1	0.10	−11.08	−5.94
2	−0.14	−11.28	−5.92
3	−0.06	−11.22	−5.86
4	−0.17	−10.62	−5.87
**Axis of rotation**			
**Rep**	**Displacement** (**mm**)	**Rotation** (**deg°**)	**Vector** **X/Y/Z**
1	0.40	43.23	−0.77**/**0.15**/**−0.23
2	0.39	43.40	−0.77**/**0.15**/**−0.23
3	0.44	43.62	−0.77**/**0.15**/**−0.23
4	0.38	43.53	−0.77**/**0.15**/**−0.23

Rep: repetition; Vector: orientation vector describing the orientation of the axis; X: dorsiflexion-plantarflexion/medio-lateral; Y: pronation-supination/anterior-posterior; Z: abduction-adduction/cranio-caudal.

### Differences between scenarios

Differences over the complete motion detected for rotation around the X-, Y- and Z- axes between Scenario 1 (Intact) and Scenario 2 (section of ATFL) ranged from 4.88° to 6.07°. Values concerning the translations along the axes varied from 0.56 mm to 2.61 mm.

Comparing motion between Scenario 1 and Scenario 3 (section of ATFL and CFL), rotation angle differences varied from 5.46° to 8.82°. The translations for this comparison varied between 0.78 mm and 2.41 mm.

The comparison of motion between Scenario 1 and Scenario 4 (section of the ATFL, CFL and PTFL), showed rotation angle differences varying from 6.93° to 10.01°. Displacement differences ranged between 0.21 mm and 3.85 mm for the talocrural joint.

**Figure 4:**
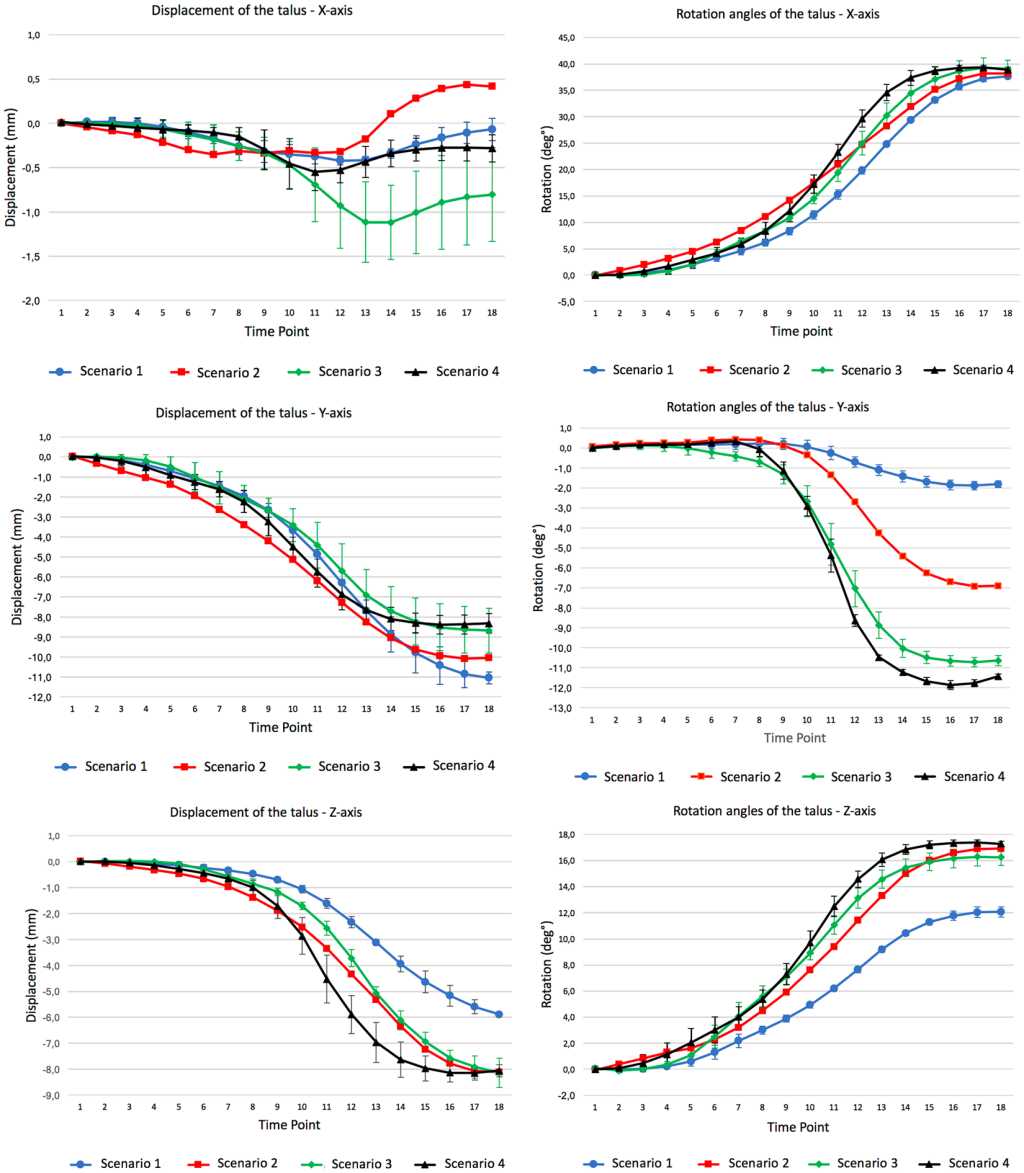
Rotation angles (degrees) and translations (mm) of the scenarios for the talocrural joint. Each curve represents the mean of four repetitions. Error bars represent the standard deviation from the mean of the four repetitions of each scenario. Scenario 1: Intact; Scenario 2: section of ATFL; Scenario 3: section of ATFL and CFL; Scenario 4: section of ATFL, CFL and PTFL.

Table 2 summarises these results and a graphical representation of the rotation and displacement values at the level of the talocrural joint at the different time points of the movement is provided in Fig. 4.

**Table 2:** Maximum differences between scenarios over the course of the movement.

Rotation angles						
	X (deg°)	%	Y (deg°)	%	Z (deg°)	%
Scenario 1 VS Scenario 2	6.07	+16%	5.12	+260%	4.88	+40%
Scenario 1 VS Scenario 3	5.46	+15%	8.82	+448%	5.47	+44%
Scenario 1 VS Scenario 4	9.83	+26%	10.01	+508%	6.93	+56%
Scenario 2 VS Scenario 3	−3.61	−25%	4.77	+117%	1.86	+17%
Scenario 2 VS Scenario 4	6.22	+22%	6.38	+156%	3.32	+30%
Scenario 3 VS Scenario 4	4.68	+19%	1.61	+18%	1.50	+10%
**Translations**						
	**X (mm)**	**%**	**Y (mm)**	**%**	**Z (mm)**	**%**
Scenario 1 VS Scenario 2	0.56	+327%	1.54	+14%	2.61	+44%
Scenario 1 VS Scenario 3	0.78	+458%	−2.37	−21%	2.41	+40%
Scenario 1 VS Scenario 4	0.21	+125%	−2.73	−24%	3.85	+65%
Scenario 2 VS Scenario 3	1.28	+459%	−1.78	−29%	−0.81	−32%
Scenario 2 VS Scenario 4	0.71	+163%	−1.73	−17%	1.65	+32%
Scenario 3 VS Scenario 4	−0.78	−70%	1.33	+30%	2.17	+58%

X: dorsiflexion-plantarflexion/medio-lateral; Y: pronation-supination/anterior-posterior; Z: abduction-adduction/cranio-caudal; Scenario 1: Intact; Scenario 2: section of ATFL; Scenario 3: section of ATFL and CFL; Scenario 4: section of ATFL, CFL and PTFL; %: indicates the percentage increase/decrease of motion relative to Scenario 1 for the first 3 comparisons, relative to Scenario 2 in the 4^th^ and 5^th^ and relative to Scenario 3 in the last comparison.

**Figure 5:**
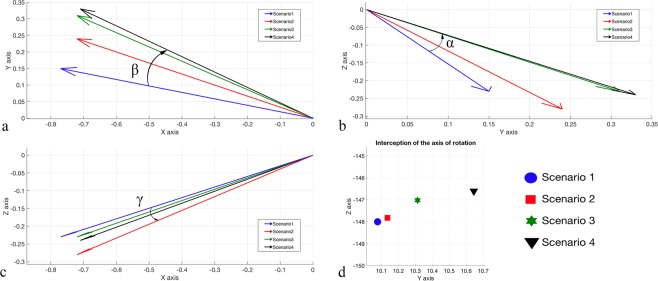
Changes in the axis of rotation for the different scenarios. Each scenario is the average of 4 repetitions. Changes in orientation of the axis of rotation for the ZY plane (**a**), XY (**b**) and XZ (**c**). (**d**) Differences in the intersection with the ZY plane passing through the centre of the articular surface of the talus; Scenario 1: Intact; Scenario 2: section of ATFL; Scenario 3: section of ATFL and CFL; Scenario 4: section of ATFL, CFL and PTFL; α angle: scenario 1 vs scenario 4; β angle: scenario 1 vs scenario 2; γ angle: scenario 1 vs scenario 4.

The axis of rotation had a maximum change in orientation between the intact scenario and the others of 20.6° (α angle Fig. 5b) on the YZ plane, 13.7° (β angle Fig. 5a) on the XY plane and 4.1° (γ angle Fig. 5c) on the XZ plane. A progressive displacement of the axis up to 4.13 mm in postero-cranial direction was observed (Fig. 5d). Table 3 shows the single components of each axis for orientation and plane intersection.

**Table 3:** Orientation and position of the axis of rotation.

	Orientation X-component	Orientation Y-component	Orientation Z-component	Interception X-axis	Interception Y-axis	Interception Z-axis
Scenario 1	−0.77	0.15	−0.23	11.46	10.08	−148.00
Scenario 2	−0.72	0.24	−0.28	11.46	10.46	−147.08
Scenario 3	−0.72	0.31	−0.23	11.46	11.58	−144.87
Scenario 4	−0.71	0.33	−0.24	11.46	11.62	−144.16

Orientation X, Y and Z: orientation vector components describing the orientation of the axis. Interception X, Y and Z: Interception of the axis of rotation with a plane parallel to the YZ plane, passing through the centre of the talus. Scenario 1: Intact; Scenario 2: section of ATFL; Scenario 3: section of ATFL and CFL; Scenario 4: section of ATFL, CFL and PTFL.

## Discussion

The current study showed that 4DCT can be used to evaluate joint kinematics and detect changes after ligaments section. Rotation angle differences between repetitions around the X-, Y- and Z- axes were below 1° and below 1 mm for displacement values. Such small differences together with a consistent orientation of the axis of rotation, highlight the repeatability of the setup used in this study.

A similar trend was shown for all kinematic parameters for almost all axes, showing a progressive increase of motion amplitude for the different scenarios. At talocrural joint level, an increased supination (Y-axis) up to five times compared to the intact motion was observed. Also, an increase in plantar flexion up to 26% (X-axis) and adduction up to 56% (Z-axis) was seen.

Regarding translations, the talocrural joint showed a gradual increase up to 65% of the distance between the two joint surfaces (cranio-caudal displacement along the Z-axis) and a reduction (−24%) of posterior-to-anterior displacement (Y-axis). Although high percentage increase of medio-lateral displacement was reported, the actual displacement remains very small. These kinematic modifications, based on Cardan angles and translations, caused a different orientation and position of the axis of rotation of the talus after ligament sectioning. Looking only at the beginning and end of the movement, it would not always have been possible to clearly distinguish the four scenarios. Contrary to this, the analysis of differences in movement during the motion showed clear changes between the intact ankle and after ligament section. One clear example is the rotation around the x-axis, where all four scenarios end in a very close position. However, it was still possible to distinguish between scenario 1 and the others considering the evolution over time. Maximum difference for rotational values around the X and Z axes were found during the movement and not at the end point. Only rotation around the Y axis showed maximum difference at the end of the movement. A possible explanation is that at the end of the motion (maximum ankle inversion) maximum stress in that direction was induced on the joint. This is very relevant for pathologies that may only take place during motion, such as trigger syndromes, which would not be detectible at the extreme of the movement.

In a similar study, Teixeira *et al*. (2017) tried to reproduce joint instability in the subtalar joint by cutting the cervical ligament of the ankle and the interosseous talo-calcaneal ligament. It was found that 4DCT could detect small changes in joint amplitude.

These preliminary findings may support the evidence that 4DCT is of added value in evaluating joint pathologies such as instabilities associated with ligamentous damage. Moreover, based on the findings of the current study it seems feasible to detect small changes during dynamic acquisition. These findings are in line with earlier studies on the wrist and thumb joint where 4DCT detected differences from below 1° and 1 mm^11,12^.

Besides kinematic analysis, radiation dose must also be considered. The current study showed an effective dose of 0.005 mSv (1.9 mGy CTDIvol) which is lower than Teixeira *et al*. (2017), who reported a dose of 0.01 mSv in a study on the subtalar joint. Considering the fact that yearly background radiation varies from 2 to 3 mSv, these values are deemed acceptable for using 4DCT *in vivo*^11,12,22–24^.

The current study had some limitations. First, kinematics of an *in vitro* setup may not exactly reflect normal physiological movements of a living subject due to the absence of muscle tone. However, the movement was externally controlled in this study to increase the reproducibility and to avoid a high radiation dose in a living subject due to the high number of repetitions. Moreover, the ligaments section would not be possible in living subjects, allowing only to examine the current kinematics without comparison of the previous (healthy) condition. The purpose of the study was not to highlight the effects of ligament section but the ability of 4DCT to detect changes, as such an *in-vitro* setup was ideal for the scope of the research. Secondly, only one specimen was available for this study, limiting the statistical analysis and any further generalisation or interpretation of the results in a clinical prospective. Third, the movement was manually induced, making the setup susceptible to errors due to a possible increase of operator-induced variability. However, comparison of repetitions showed that the setup was reliable with differences smaller than 1 mm and 1° between repetitions. Moreover, variability of the four repetitions for each scenario was considered when maximum values were calculated. Fourth, the study was not performed in weight bearing, which would represent a more functional position for the lower limb. At the current state, no device that combines weight bearing and motion during CT acquisition is available. Last, the skin dose was not measured in this study. Dynamic CT acquisition is based on scanning the same area over a period of time and even in the presence of a high skin dose, the effective dose can remain low. Thus, although our study reported very low CTDI_vol_ showing a low radiation exposure, the skin dose may be a more sensitive parameter to determine radiation exposure and should be measured in further clinical studies.

This study demonstrated that kinematic changes in the talocrural joint, due to lateral collateral ankle ligament failure, can be detected with 4DCT *in vitro* with a minimum amount of radiation exposure. Sequential sectioning of the lateral collateral ankle ligaments progressively increased the angular motion and changed the amount and direction of bony displacement. The change in orientation of the axis of rotation was also highlighted. It was also shown that more ligamentous damage induced more changes in kinematics compared with an intact scenario. Due to the high temporal and spatial resolution of 4DCT, detailed acquisition of *in vivo* situations is possible. As such, this method can be used to investigate pathologies that present themselves predominantly during motion. Quantifying the amount of lateral collateral ankle ligament damage and kinematics patterns could have future applications in clinical practice. If the kinematics analysis show predominant pattern alterations it may be more appropriate to focus on an intervention that aims to correct the trajectory of the movement (bracing, taping, balance training). On the other hand, if the magnitude of movement is altered, other interventions such as manual treatment (improve motion amplitude) or surgical intervention (reduce joint instability) may be more appropriate. However, further studies with bigger *in vitro* sample sizes and consequent investigations in living subjects are recommended to confirm the current findings and offer reference values suitable for clinical practice.

## References

[1] Gondim Teixeira PA (2017). Evidence-based recommendations for musculoskeletal kinematic 4D-CT studies using wide area-detector scanners: a phantom study with cadaveric correlation. Eur. Radiol..

[2] Kerkhof FD (2016). Quantifying thumb opposition kinematics using dynamic computed tomography. J. Biomech..

[3] Tay SC (2007). Four-dimensional computed tomographic imaging in the wrist: Proof of feasibility in a cadaveric model. Skeletal Radiol..

[4] Teixeira PAG (2015). Musculoskeletal Wide-Detector CT Kinematic Evaluation: From Motion to Image. Semin. Musculoskelet. Radiol..

[5] De Aguiar TO (2017). Simultaneous Radiographic Technique to Evaluate Ankle Instability. Arthrosc. Tech..

[6] Dowling LB, Giakoumis M, Ryan JD (2014). Narrowing the normal range for lateral ankle ligament stability with stress radiography. J. Foot Ankle Surg..

[7] Hoffman E (2011). Accuracy of Plain Radiographs Versus 3D Analysis of Ankle Stress Test. Foot Ankle Int..

[8] Teixeira PAG (2017). Quantitative analysis of subtalar joint motion with 4D CT: Proof of concept with cadaveric and healthy subject evaluation. Am. J. Roentgenol..

[9] Mat Jais IS, Tay SC (2017). Kinematic analysis of the scaphoid using gated four-dimensional CT. Clin. Radiol..

[10] Leng S (2011). Dynamic CT technique for assessment of wrist joint instabilities. Med. Phys..

[11] Zhao K (2015). A Technique for Quantifying Wrist Motion Using Four-Dimensional Computed Tomography: Approach and Validation. J. Biomech. Eng..

[12] Goto A (2014). *In vivo* pilot study evaluating the thumb carpometacarpal joint during circumduction. In. Clinical Orthopaedics and Related Research.

[13] Demehri S (2015). Evaluation of pisotriquetral motion pattern using four-dimensional CT: Initial clinical experience in asymptomatic wrists. Clin. Radiol..

[14] Arab, W. A. *et al*. Scapholunate instability: improved detection with semi-automated kinematic CT analysis during stress maneuvers. *Eur*. *Radiol* (2018). 10.1007/s00330-018-5430-2 29713765

[15] Yoo TS (2002). Engineering and algorithm design for an image processing API: A technical report on ITK - The Insight Toolkit. In. Studies in Health Technology and Informatics.

[16] Yushkevich PA (2006). User-guided 3D active contour segmentation of anatomical structures: Significantly improved efficiency and reliability. Neuroimage.

[17] Klein S, Staring M, Murphy K, Viergever MA, Pluim JPW (2010). Elastix: A toolbox for intensity-based medical image registration. IEEE Trans. Med. Imaging.

[18] Shamonin DP (2014). Fast parallel image registration on CPU and GPU for diagnostic classification of Alzheimer’s disease. Front. Neuroinform..

[19] Cheung, W. & Hamarneh, G. N-sift: N-dimensional scale invariant feature transform for matching medical images. *4th IEEE International Symposium on* Biomedical *Imaging: From Nano to Macro*, 720–723 (2007).

[20] Fitzpatrick JM, West JB (2001). The distribution of target registration error in rigid-body point-based registration. IEEE transactions on medical imaging.

[21] Spoor CW, Veldpaus FE (1980). Rigid body motion calculated from spatial co-ordinates of markers. J. Biomech..

[22] UNSCEAR. United Nations Scientific Committee on the Effects of Atomic Radiation. *Sources and Effects of Ionizing Radiation*. *UNSCEAR* 2008 *Report* **1** (2000).

[23] Biswas D (2009). Radiation exposure from musculoskeletal computerized tomographic scans. J. Bone Jt. Surg. - Ser. A.

[24] Thurston J (2010). NCRP Report No. 160: Ionizing Radiation Exposure of the Population of the United States. Phys. Med. Biol..

